# Identification of Novel *BRCA1* and *RAD50* Mutations Associated With Breast Cancer Predisposition in Tunisian Patients

**DOI:** 10.3389/fgene.2020.552971

**Published:** 2020-11-06

**Authors:** Najah Mighri, Yosr Hamdi, Maroua Boujemaa, Houcemeddine Othman, Sonia Ben Nasr, Houda El Benna, Nesrine Mejri, Soumaya Labidi, Jihen Ayari, Olfa Jaidene, Hanen Bouaziz, Mariem Ben Rekaya, Ridha M’rad, Abderrazek Haddaoui, Khaled Rahal, Hamouda Boussen, Samir Boubaker, Sonia Abdelhak

**Affiliations:** ^1^Laboratory of Biomedical Genomics and Oncogenetics, LR16IPT05, Institut Pasteur de Tunis, University of Tunis El Manar, Tunis, Tunisia; ^2^Sydney Brenner Institute for Molecular Bioscience, University of the Witwatersrand, Johannesburg, South Africa; ^3^The Military Hospital of Tunis, Department of Medical Oncology, Tunis, Tunisia; ^4^Department of Medical Oncology, Abderrahmane Mami Hospital, Ariana, Tunisia; ^5^Department of Carcinological Surgery, Salah Azaiez Institute, Tunis, Tunisia; ^6^Service des Maladies Congénitales et Héréditaires, Hôpital Charles Nicolle, Tunis, Tunisia

**Keywords:** breast cancer, genetic screening, *BRCA1*-founder mutation, next-generation sequencing, novel mutation, *RAD50*

## Abstract

**Background:**

Deleterious mutations on *BRCA1/2* genes are known to confer high risk of developing breast and ovarian cancers. The identification of these mutations not only helped in selecting high risk individuals that need appropriate prevention approaches but also led to the development of the PARP-inhibitors targeted therapy. This study aims to assess the prevalence of the most frequent *BRCA1* mutation in Tunisia, c.211dupA, and provide evidence of its common origin as well as its clinicopathological characteristics. We also aimed to identify additional actionable variants using classical and next generation sequencing technologies (NGS) which would allow to implement cost-effective genetic testing in limited resource countries.

**Patients and Methods:**

Using sanger sequencing, 112 breast cancer families were screened for c.211dupA. A set of patients that do not carry this mutation were investigated using NGS. Haplotype analysis was performed to assess the founder effect and to estimate the age of this mutation. Correlations between genetic and clinical data were also performed.

**Results:**

The c.211dupA mutation was identified in 8 carriers and a novel private *BRCA1* mutation, c.2418dupA, was identified in one carrier. Both mutations are likely specific to North-Eastern Tunisia. Haplotype analysis supported the founder effect of c.211dupA and showed its recent origin. Phenotype-genotype correlation showed that both *BRCA1* mutations seem to be associated with a severe phenotype. Whole Exome Sequencing (WES) analysis of a *BRCA* negative family revealed a Variant of Unknown Significance, c.3647C > G on *RAD50*. Molecular modeling showed that this variant could be classified as deleterious as it is responsible for destabilizing the RAD50 protein structure. Variant prioritization and pathway analysis of the WES data showed additional interesting candidate genes including *MITF* and *ANKS6.*

**Conclusion:**

We recommend the prioritization of *BRCA1*-c.211dupA screening in high risk breast cancer families originating from the North-East of Tunisia. We also highlighted the importance of NGS in detecting novel mutations, such as *RAD50-*c.3647C > G. In addition, we strongly recommend using data from different ethnic groups to review the pathogenicity of this variant and reconsider its classification in ClinVar.

## Introduction

Breast cancer remains the most common cancer among women worldwide (Bray et al., 2018). Each year, 2000 new breast cancer cases are diagnosed in Tunisia (Bray et al., 2018) and the mean age at diagnosis is around 50 years old, a decade younger than Western countries (Chalabi et al., 2008).

*BRCA1* and *BRCA2* are the most studied breast cancer susceptibility genes that explain a significant proportion of hereditary breast and ovarian cancers (Miki et al., 1994; Wooster et al., 1995; Ford et al., 1998). It has been reported that the average cumulative risks in *BRCA1*-mutation carriers by age of 70 were 65% and 39% for breast and ovarian cancers respectively and the corresponding estimates for *BRCA2* were 45% and 11% (Antoniou et al., 2003). The frequency of germline mutations identified on the *BRCA* genes varies depending on the geographic and the ethnic distributions. Indeed, while most variations are shared between human populations, some of the *BRCA1/2* mutations are ethnic specific (Fackenthal and Olopade, 2007).

Since a good knowledge of population structure is essential for the development of efficient and individualized screening approaches, the identification of founder *BRCA* mutations is crucial to set up affordable and cost-effective breast cancer genetic testing. In North Africa, several *BRCA1/2* founder mutations were identified among which *BRCA1*-c.798_799delTT that was reported in 9.8% and 36.4% of familial and sporadic Algerian breast cancer cases, respectively (Uhrhammer et al., 2008). This same mutation was reported in Tunisian population with a frequency of 18% (Mahfoudh et al., 2012). Furthermore, *BRCA2-*c.1310_1313delAAGA is considered as a North African founder mutation since it has been identified in Algerian, Moroccan and Tunisian breast cancer cases (Cherbal et al., 2010; Fourati et al., 2014; Laarabi et al., 2017).

In Tunisia, founder mutations explain around 42% of hereditary disorders (Romdhane et al., 2012). This is mainly due to the high rates of consanguinity and endogamy. So far, few *BRCA1* deleterious mutations were identified in Tunisia and the most recurrent ones are c.211dupA and c.5266dupC (Troudi et al., 2008; Mahfoudh et al., 2012; Fourati et al., 2014; Riahi et al., 2015). *BRCA1*-c.5266dupC was originally described as an Ashkenazi founder mutation. Then, it has been reported in several other populations such as Russian, Italian, Slovenian, Greek and Tunisian (Janavičius, 2010). However, the c.211dupA mutation seems to be specific to Tunisia since it has never been previously described in any other population (Troudi et al., 2007).

Besides *BRCA1* and *BRCA2*, more than 100 loci are known to be associated with breast cancer risk (Han et al., 2016). For *BRCA* negative families, fine mapping of other high to moderate penetrant breast cancer genes is needed to explain the genetic predisposition to breast cancer. For this purpose, next generation sequencing technologies are widely used as a cost-effective approach for the detection of novel mutations (Shahi et al., 2019). Indeed, target gene sequencing (TGS), whole exome sequencing (WES), and whole genome sequencing (WGS) are the main protocols currently used to identify either new mutations or new candidate genes associated with hereditary disorders (Chandler et al., 2016; Akter et al., 2019; Bewicke-Copley et al., 2019; Posey, 2019; Shahi et al., 2019).

In the present study, we used classical as well as next generation sequencing technologies to investigate the genetic predisposition to breast cancer in 112 Tunisian families. Targeted mutation screening followed by target gene and whole exome sequencing were performed. We have also studied the founder effect and the relative age of the most frequent *BRCA1* mutation in Tunisia (c.211dupA). In addition, genotypic data were correlated to drug response, clinicopathological and phenotypic features of all mutation carriers.

## Materials and Methods

### Patients

A total of 122 individuals with strong familial history of breast cancer (111 patients and 11 healthy) were recruited from three principal medical oncology centers based in Tunis that provide care and services for Tunisian cancer patients from all over the country (Departments of Medical oncology of Abderrahmane Mami Hospital, of Military Hospital of Tunis and the surgical oncology Department of Institut Salah Azaiez). Prior to specimen collection, all participants signed written and informed consents and gave relevant information about their personal and familial cancer histories as well as their geographic origins. A patient was recruited if at least one of the following criteria is fulfilled: (1) Presence of at least two related first or second-degree breast cancer cases, (2) Breast cancer in young patients aged less than 36 years, (3) Presence of at least two cases of breast or ovarian cancer, regardless of age. The mean age at diagnosis of the whole studied cohort is 42.84 years. The geographic distribution of birth locations is as follows; the majority are originating from Northern Tunisia, however approximately 20% are from the Central region and 6% from the South region. Forty-three percent of patients have a strong family history of breast cancer where 2 or multiple family members were diagnosed with breast cancer. A family history of ovarian cancer was observed among approximately 10% of patients. Breast cancer patients selected for next generation sequencing are those with the strongest family history of breast cancer (having 2 or more cases) and/or with age at onset of less than 36 years.

Clinico-pathological characteristics and follow-up data have been collected from medical records of patients.

### Sanger Sequencing

Total genomic DNA was isolated from peripheral blood using DNeasy blood DNA extraction Kit (Qiagen) according to the manufacturer’s instructions. DNA purity and concentration were measured using a NanoDrop^TM^ spectrophotometer.

Polymerase chain reaction reactions were performed on genomic DNA, following standard protocols. Sanger sequencing was performed using an automated sequencer (ABI 3500; Applied Biosystems, Foster City, CA, United States) and a cycle sequencing reaction kit (Bigdye Terminator v3.1 kit, Applied Biosystems). Data was analyzed using BioEdit software version 7.2.5.

Sanger sequencing technique was first used to screen the c.211dupA mutation on exon 5 of *BRCA1* (NM_007294.3) among all 122 participants. It was also used to validate the identified variants resulting from the NGS assays. Non-carriers of c.211dupA mutation were tested for other recurrent *BRCA1/2* mutations in Tunisia namely *BRCA1-*c.798_799delTT, *BRCA1-*c.5266dupC, and *BRCA2-*c.1310_1313delAAGA (Mahfoudh et al., 2012; Fourati et al., 2014; Msolly and Kassab, 2015; Riahi et al., 2015).

### Targeted *BRCA1/2* Genes Sequencing

Targeted *BRCA1/2* genes sequencing was performed for 9 patients. A DNA quantity of 40 ng measured by Qubit (Thermo Fisher Scientific, MA, United States) was used as a template to generate libraries for sequencing. Libraries were prepared using the QIAGEN Library Kit v2.0 and the GeneRead QIAact *BRCA1/2* panel (QIAGEN, Hilden, Germany), which comprises 253 pooled primer pairs custom designed to cover all coding regions of the *BRCA1* and *BRCA2* genes, including 20 bp flanking regions in adjacent introns. All steps of library preparation were performed according to the manufacturer’s protocol. The libraries were then quantified using a Qubit dsDNA HS Assay Kit (Life Technologies, MA, United States) and QIAxcel (QIAGEN, Hilden, Germany). Nine individual libraries were pooled prior to emulsion PCR and bead enrichment steps that were carried out using an automated protocol on the GeneRead QIAcube (QIAGEN, Hilden, Germany) using the GeneRead Clonal Amp Q Kit (QIAGEN, Hilden, Germany), according to the manufacturer’s protocol. Following bead enrichment, the pooled libraries were sequenced using the GeneReader platform (QIAGEN, Hilden, Germany).

### Whole Exome Sequencing (WES)

Whole exome sequencing was performed for 8 patients. Samples were prepared according to Agilent SureSelect Protocol Version 1.2 and enrichment was carried out according to Agilent SureSelect protocols. Sequencing was performed on Illumina HiSeq2000 platform using TruSeq v3 chemistry with paired-end (2 × 100 pb). Exome DNA sequences were mapped to their location based on the hg19/b37 human genome using the Burrows–Wheeler Aligner (BWA) package (Li and Durbin, 2009). Duplicate reads were removed using Picard (Ebbert et al., 2016). The subsequent SAM files were converted to BAM files using Samtools (Li et al., 2009). GATK was then used to recalibrate the base quality scores as well as for SNP and short INDEL calling (McKenna et al., 2010).

### Functional Annotation and Variants Prioritization

Annotation and prioritization of potential disease-causing variants were performed using VarAFT (Variant Annotation and Filtering Tool) 2.10 software (Desvignes et al., 2018). The annotated InDels and SNPs were filtered according to several criteria: (1) considering breast cancer as autosomal dominant disease, we removed variants that were found at homozygous state, (2) variants identified as intronic, intergenic and non-coding or synonymous were discarded, (3) assuming that causal variants are rare, we removed all variants with an allele frequency > 1% either in 1000 Genomes or GnomAD_Exome databases, (4) benign or tolerated variants were discarded according to the following *in silico* prediction tools: Sorting Intolerant From Tolerant (SIFT), PolyPhen-2, Mutation Taster, LRT_pred and Combined Annotation Dependent Depletion CADD tool. We used SiPhy_log odds score calculated by ANNOVAR to keep deleterious variants with high scores and Gene Damage Index Prediction database (GDI) to exclude highly mutated genes.

The Wikipathways (Slenter et al., 2017) and Kyoto Encyclopedia of Genes and Genomes (KEGG) (Kanehisa and Goto, 2000) databases were used to assign genes to their pathways and the Network of Cancer Genes V 6.0 (Repana et al., 2019) was used to identify genes associated with cancers.

### Molecular Modeling

We used the protein sequence of RAD50 annotated under the accession Q92878 in the Uniprot database (The Uniport Consortium, 2019) as a reference sequence. To predict the 3D structure of the wild type RAD50 we used a comparative modeling approach with the satisfaction of spatial restraints using MODELLER version 9.22 (Šali and Blundell, 1993). Template identification consists of searching for homologous proteins to the RAD50 reference sequence in the Protein Data Bank (PDB) (Berman et al., 2000) using BLAST (identity sequence cutoff > 30%) (Altschul et al., 1990).

The search for a template identified the structure of “DH domain-containing protein” from *Chaetomium thermophilum* (PDB code 5DAC) (Seifert et al., 2016) that shows a significant e-value of 4.2 e^–72^ and a sequence identity of 50% (locally aligned) compared to hRAD50. The alignment of hRAD50 with the template covers 32% of the reference sequence including the position of the mutation. We introduced a distance restraint of 10 Å between the terminal residues of the coiled-coil segments belonging to the same monomer. Moreover, we built the model in the presence of ATPγS: Mg^2+^ heteroatoms.

The template and target sequences were then aligned using ‘needle’ from the EMBOSS package (Rice et al., 2000) and the alignment was then edited manually to correct for errors. We then built the model by generating 50 conformers with different seeds using a rapid refinement protocol. The final model was selected based on the calculation of the DOPE score (Shen and Sali, 2006) and the quality assessment was checked by establishing the Ramachandran plot (Lovell et al., 2003) and the local energy profile Verify3D (Lüthy et al., 1992). The structure of the mutant p.Ala1216Gly was then built using the 3D model of the wild type based on MODELLER script that mutates and refines the protein structure.

### Stability Analysis

We estimated the free energy of folding between the wild type and the mutant structure (ΔΔG_Wt__–__Mut_) using DynaMut (Rodrigues et al., 2018). Moreover, we performed a simulation of protein flexibility with CABS-flex 2.0 (Jamroz et al., 2014) for both the wild type and the mutant structures at a temperature of 1.6 (dimensionless value related to the physical temperature). For each protein, we performed four simulations with four different predefined seeds. The same set is used for the wild type and the mutant 3D structures. We run the simulation with a fully flexible backbone (protein-flexibility = 0), a number of Monte Carlo cycles of 100 (*y* = 100) and the protein restraints were reduced by a factor of 0.8. Root Mean Square Fluctuation (RMSF) value was analyzed by calculating the per residue one-tailed Welch’s *t*-test under the null hypothesis that the RMSF at a given position in the sequence does not differ between the mutant and the wild type and an alternative hypothesis being that the RMSF value at given position of the sequence is greater for the mutant than the wild type structure. The analysis was performed using an in-house python script that employs the Scipy library for scientific computing.

### Copy Number Variations Analysis

Copy Number Variations (CNVs) calling was performed on WES data using the ExomeDepth R package (Plagnol et al., 2012). All CNVs were annotated using AnnotSV software (Geoffroy et al., 2018), a program designed for annotating and ranking Structural Variations (SV) from genomes. This tool compiles functionally, regulatory and clinically relevant information and aims to provide useful annotations to interpret SV potential pathogenicity. AnnotSV classifies CNVs into 5 classes (Class 1: benign, Class 2: likely benign, Class 3: VUS (Variant of Unknown Significance), Class 4: likely pathogenic, and Class 5: pathogenic). In order to identify the most relevant CNVs which may be associated with breast cancer risk, only CNVs classified as class 3, 4, and 5 were kept for further analysis. Biological pathways analysis has been performed using Wikipathways (Slenter et al., 2017) and KEGG (Kanehisa and Goto, 2000) datasets. Network of Cancer Genes V6.0 (Repana et al., 2019) was used to identify genes associated with cancers.

### Genotyping and Haplotype Analysis of c.211dupA Mutation

Haplotype analysis was carried out for 39 individuals: six carriers of the c.211dupA mutation, 3 relatives, 15 breast cancer non-carriers, and 15 healthy age-matched Tunisian women from the general population. Four microsatellite markers (D17S800, D17S855 D17S902, and D17S806), spanning a 6.75 Mb region around the *BRCA1* gene were studied (Supplementary Figure 1). Primers’ sequences were obtained from the Probe NCBI database and PCR conditions are available on request. PCR product size was evaluated by capillary electrophoresis on an ABI prism 3500 DNA Genetic Analyzer using the GeneMapper V.5.0 software (Applied Biosystems, Foster City, CA, United States). For the D17S806 marker, results were inconclusive, and it was excluded from further analysis. Haplotypes were then reconstructed using PHASE v.2.1 software (Stephens et al., 2001) that uses Bayesian methods to predict haplotype distribution.

### Estimation of c.211dupA Mutation Age

The DMLE + 2.3 program (Reeve and Rannala, 2002) was used to estimate the age of the c.211dupA mutation. The DMLE input file included the full genotypes or haplotypes of probands and controls for the analyzed markers, chromosome map distances derived from the Marshfield sex-average genetic map, population growth rate per generation and an estimate of the proportion of disease chromosomes sampled. The other DMLE parameters were kept to their default values.

For the population growth rate (r), we used data from the Tunisian Institute of Statistics (INS^1^). This key parameter was estimated to be 1.348 per generation using the following formula: p_1_ = p_0_ × r^g^, where p_1_ represents the Tunisian population size in 2014 (10.982.754), p_0_ represents the Tunisian population in 1860 (1.1 million) (Seklani, 1974), and g is the number of generations between these two-time points.

## Results

### Investigating the State of *BRCA1*-c.211dupA, Founder Effect and Age

In the current study, 122 individuals from 112 Tunisian families with strong familial history of breast cancer were investigated using sanger and next generation sequencing. First, we looked for c.211dupA which is the most recurrent *BRCA1* mutation in the Tunisian population.

A total of 8 carriers of c.211dupA belonging to 5 unrelated families were identified. All carriers originated from the North-Eastern region of Tunisia. The relative frequency of this mutation is 12.2% (5 out of 41) and 4.46% (5 out of 112) in this region and in the whole cohort, respectively. *BRCA1*-c.211dupA seems to be the most frequent *BRCA* mutation in Tunisia, accounting for approximately 41% of all identified *BRCA1* mutations, if we consider this report and previous Tunisian studies (Troudi et al., 2007; Fourati et al., 2014; Riahi et al., 2015). In addition, we have not been able to identify any of the other recurrent mutations (*BRCA1*-c.798_799delTT, *BRCA1*-c.5266dupC, and *BRCA2*-c.1310_1313delAAGA) in the other non-carrier patients of the c.211dupA mutation.

To investigate the founder effect of c.211dupA, haplotype analysis was performed for 39 individuals. Results showed that 5 out of the 6 genotyped carriers shared an identical haplotype (170-145-147), corresponding to D17S800, D17S855, and D17S902 respectively and all carriers shared a common semi-haplotype (170-145) spanning a region of 2.15 Mb (1.08cM) on *BRCA1*. This haplotype was absent in non-carriers and was not found in control chromosomes. These results support the founder effect of the c.211dupA mutation in the North-East of Tunisia.

Given that the prevalence of *BRCA1* and *BRCA2* carriers in the Tunisian general population is not well defined, for the mutation age estimation, three separate analyses were performed, each using a different estimate for the proportion of sampled population carrying the c.211dupA mutation: 0.005, 0.01, and 0.015. Thus, the age estimates for c.211dupA mutation were approximately 6.5 generations (95% CI [5.2–8.8]), 6.3 generations (95% CI [4.7–8.5]), and 6.00 generations (95% CI [4.4–8.1]). Assuming a generation time of 20 years, the corresponding ages are 130 (95% CI [104–176]), 126 (95% CI [94–170]), and 120 (95% CI [88–162]) years.

### Clinico-Pathological Features and Follow-up of c.211dupA Mutation Carriers

We further extended our analysis to the 9 additional carriers of c.211dupA mutation reported in previous Tunisian studies (Troudi et al., 2007; Fourati et al., 2014; Riahi et al., 2015). For a total of 15 c.211dupA carriers, the mean age at diagnosis was 39.41 years, ranging from 28 to 58 years (Table 1). Family history of breast cancer was observed in 93.33% cases (14/15) among which 20% (3/15) presented a family history of ovarian cancer. Other malignancies such as leukemia, prostate, colon and cervical cancers were observed in family members. Infiltrating ductal carcinoma (IDC) was the predominant tumor subtype, high grade 3 and axillary node involvement were observed in about 50% of carriers. The mean tumor size was 30 mm and 50% of carriers displayed a triple negative breast cancer (TNBC) phenotype with two additional cases harboring negative hormone receptors with unknown human epidermal growth factor receptor 2 (HER2) status. Thus, this mutation is likely associated with a severe phenotype.

**TABLE 1:** Clinico-pathological features of *BRCA1* and *RAD50* carriers.

Carrier ID	Pathology	Age at diagnosis (years)	Family History BC/OC	Family history of other cancers	Histological subtype	SBR grade	ER status	PR status	HER2 status	Nodal status	Tumor size (mm)	Ki67-index (%)	Metastatic status	Therapy	Follow-up
BC-TN-F009	BC and OC	42, CBC at 63	1 BC/OC 1 OC	Cervical cancer	IDC	NA	NA	NA	NA	NA	NA	NA	−	MCA, Adjuvant FEC-TXT, LRR	OC 15 years after the 1st BC diagnosis. CBC after 21 years. Died 25 years after BC diagnosis.
BC-TN-F0019	BC	49	3 BC	None	IDC	II	ER −	PR −	NA	N−	60	NA	−	MCA, Adjuvant chemo- therapy, LRR	NA
BC-TN-F0049-1	BC	29 CBC at 32	2 BC	Leukemia, Prostate, Colon, Gyneco- logical, Larynx	IDC	III	ER − ER +	PR− PR−	HER2− HER2−	N + N−	35 38	NA 40	−	MCA, Adjuvant 4FEC, 4TXL-carboplatin, LRR	Spontaneous pregnancy 6 months after the end of CT. CBC at 32 years old, 3 years after the 1^ st^ BC diagnosis. Adjuvant paclitaxel- carboplatin. Follow-up of 4 years.
BC-TN-F0049-2	BC	37	2 BC	Leukemia, Prostate, Colon, Gyneco- logical, Larynx	IDC	III	ER +	PR +	HER2−	N +	15	2	−	MCA, Adjuvant 3FEC-9 weekly TXL, LRR, TAM and zoladex: 1 injection/month (18 injections)	Patient in complete remission with a follow-up of 3 years
BC-TN-F0093	BC	34	None	Lung, pancreatic	IDC	II	ER −	PR −	HER2−	NA	NA	30	NA	NA	Died with disease progression.
BC-TN-F199	BC	58	1 BC 1 OC	Endometrial, Lung	IDC	III	ER −	PR −	HER2-	N +	40	20	+ (lung, bone)	MCA, first-line CT: 6FEC, Cerebral RT,	Lung and bone metastases. Progression after 3 cycles of FEC. Second-line CT: capecitabin during 5 months with objective response after 3 cycles. Cerebral metastasis. c.211dupA *BRCA1* mutation result Third-line CT 3 weekly carboplatin Died by disease progression 1 year after BC diagnosis.
BC-TN-F204	BC and OC	28	2 BC 2 OC	Thyroid	IDC	NA	ER −	PR −	HER2−	N−	30	NA	−	TCA, Adjuvant FEC-TXT, LRR	OC 13 years after BC. Complete surgery followed by adjuvant paclitaxel-carboplatin Patient in complete remission with a follow up of 16 years.
PEC-TN-F50-1	BC	38	2 BC	Lung, Pancreatic	IDC	II	ER −	PR−	HER2−	NA	NA	30	−		Newly diagnosed

*BC, Breast Cancer; CBC, Contralateral Breast Cancer; CT, Chemotherapy; ER, Estrogen Receptor; FEC, 5-Fluorouracil-Epirubicin-Cyclophosmamide; HER2, Human Epidermal Growth Factor Receptor2; IDC, Invasive Ductal Carcinoma; LRR, LocoRegional Radiotherapy; MCA, Mastectomy; NA, Not Available; OC, Ovarian Cancer; PR, Progesterone Receptor; RT, Radiotherapy; SBR, Scarff-Bloom and Richardson; TAM; Tamoxifen; TCA, Tumorectomy; TXL, Paclitaxel; TXT, Docetaxel.*

Assessment of disease evolution for c.211dupA carriers as well as their response to treatment were performed. Radical mastectomy was performed in 4 cases. Sequential adjuvant chemotherapy, 5-Fluorouracil-Epirubicin-Cyclophosphamide-Paclitaxel/Docetaxel (FEC-TXL/TXT) was administered to the majority of carriers and radiotherapy was performed in non-metastatic cases (Table 1). Two carriers (BC-TN-F204 and BC-TN-F009) have developed ovarian cancer 13 and 15 years after their breast cancer diagnosis respectively. Contralateral recurrence was observed in two cases (BC-TN-F009 and BC-TN-F0049-1). One patient diagnosed with triple negative metastatic breast cancer (BC-TN-F199) has a refractory disease treatment and died within 12 months by disease progression. However, the median survival of non-metastatic carriers was 12 years. We therefore concluded that except for the metastatic breast cancer case, chemotherapy treatments were well tolerated, and carriers had a good survival despite the severity of their clinico-pathological phenotype.

### BRCA1 and BRCA2 Variants Identified by Next-Generation Sequencing

**FIGURE 1:**
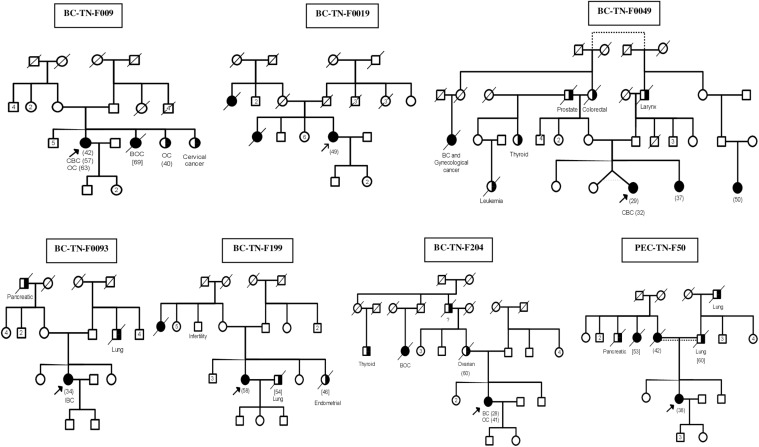
Familial Pedigrees of *BRCA1* and *RAD50* mutations carriers. Available age at diagnosis of breast cancer cases is indicated between brackets. For some cases, only age at death was available and is indicated in square brackets. BOC, Breast and ovarian cancers; CBC, Contralateral breast cancer; IBC, Inflammatory breast cancer; OC, ovarian cancer. Black boxes mean breast cancer and half black boxes mean other cancer types.

After studying the carriers of c.211dupA mutation in detail, we then selected 17 other breast cancer patients to be investigated by next generation sequencing. The selection was done on the basis of family history and disease onset. Targeted *BRCA1/2* sequencing and whole exome sequencing were performed on 9 and 8 breast cancer patients respectively. Familial pedigrees as well as phenotypic characteristics of all carriers are shown in Figure 1 and Table 1 respectively.

**FIGURE 2:**
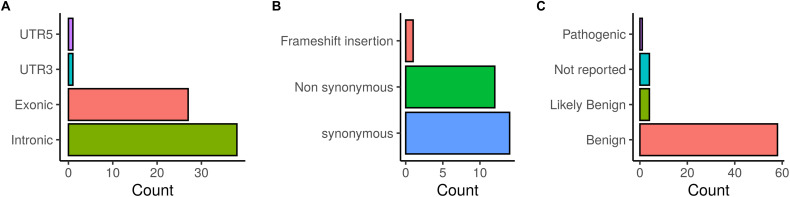
Distribution of *BRCA1/2* variations identified in patients investigated by target gene and whole exome sequencing. **(A)** SNVs/InDels distribution, **(B)** Distribution of coding variations, **(C)** ClinVar classification of detected variations.

The distribution of the identified *BRCA1/2* variations is illustrated in Figure 2. Exonic coding variants represented 27 out of 67 variants and based on ClinVar database, the majority of the identified *BRCA1/2* variants were classified as benign (58), 4 were classified as likely benign and 4 variants were not reported in ClinVar.

Additionally, our results identified a novel *BRCA1* pathogenic mutation on exon 11 (c.2418dupA and rs886040036) in one patient. This mutation has not been reported in previous studies neither in Tunisia nor in other populations. Nevertheless, it is already listed and classified as pathogenic in ClinVar and predicted to result in the substitution of Alanine to Serine (p.Ala807Serfs) followed by a premature truncation of BRCA1 protein at amino acid position 809.

Patient (BC-TN-F0093) harboring the c.2418dupA mutation is also originating from the North Eastern region of Tunisia. She was diagnosed with triple negative inflammatory breast cancer at 34 years old. Pathological examination of the tumor revealed invasive ductal carcinoma, Scarff-Bloom and Richardson (SBR) grade 2 with Ki-67 of 30%. No additional carriers of this mutation were found in the studied cohort.

The remaining 16 patients did not carry any pathogenic mutation on the *BRCA* genes.

### Whole Exome Sequencing Data Analysis

#### Analysis of Known Breast Cancer Susceptibility Genes

In our current study, in addition to the sanger sequencing analysis, and after screening for pathogenic *BRCA1/2* mutations among 17 breast cancer patients by NGS. We then selected a unique patient investigated by WES, originating from the North-East of Tunisia for detailed analysis, in order to shed light on the genetic architecture of breast cancer in this specific region. First, we looked for variations on 29 genes known to be associated with breast cancer. At this step we have identified a VUS on *RAD50*. Therefore, for this same patient, we extended our analysis in order to select other relevant variants on candidate genes that could be associated with malignancies based on *in silico* analysis and several genomic and biological pathways databases. The proband (BC-TN-F0019) was diagnosed with a locally advanced breast cancer at age 49 with negative hormone receptors status and SBR grade 2. Using whole exome data, we first investigated a list of 29 genes known to be associated with hereditary breast and ovarian cancers. Coverage analysis for these 29 genes demonstrated that we reached a depth of ∼50X and a percentage of coverage of 82% for the targeted regions (Supplementary Table 1).

A total of 31 exonic variants and one splicing SNP were identified on the 29 genes (Supplementary Table 2). None of these variants was classified as pathogenic in ClinVar. However, a VUS, c.3647C > G, p.Ala1216Gly, was identified on *RAD50*. In the same patient, another variant, rs28908468, described by ClinVar as associated with “Drug response” (PARP inhibitors), was identified on *RAD51B.*

We then made a detailed analysis of the mutation identified in the *RAD50* gene in order to investigate the possibility of revising its current classification in public databases such as ClinVar. Several *in silico* analysis, indeed, suggest the deleterious aspect of the RAD50 p.Ala1216Gly mutation.

**FIGURE 3:**
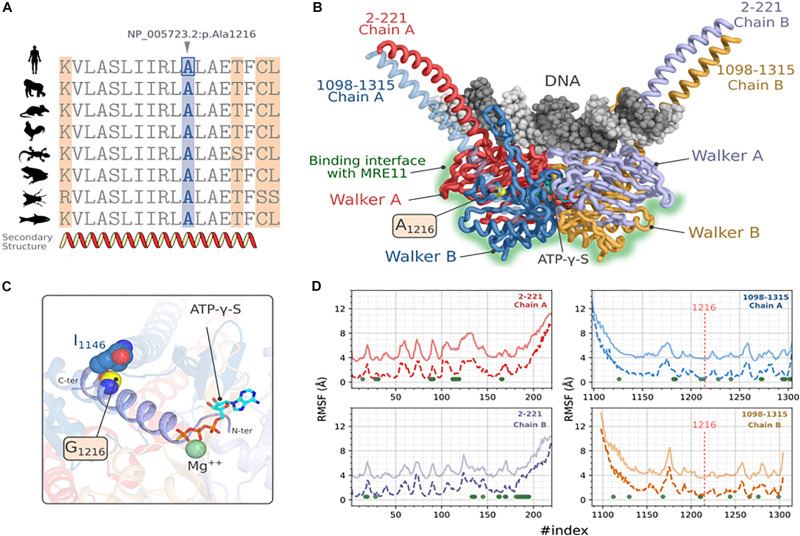
Molecular modeling and stability analysis of RAD50_p.Ala1216Gly variation. **(A)** Multiple sequence alignment of hRAD50 with homologous proteins from model organisms, *Gorilla* (XP_004042521.1), *Mus musculus* (NP_033038.2), *Gallus* (XP_414645.3), *Xenopus laevis* (NP_001154855.1), *Danio rerio* (XP_005167995.1), *Anolis carolinensis* (XP_003217435.1) and *Drosophila melanogaster* (NP_001246461.1). **(B)** Predicted 3D structure of hRAD50 predicted with comparative modeling showing the mutation site and the different domains of the protein. DNA docking was generated by transferring the coordinates of the nucleic acid after a structural superposition of the symmetric chains. **(C)** Position of the mutation A1216G on the ATPγS:Mg ++ stabilizing α-helix extended over residues 1203-1221. **(D)** Comparative RMSF analysis between the reference protein (A1216, solid lines) and the mutated protein (G1216, dashed lines). The values of RMSF for the reference protein were shifted to +3 Å. For the sake of visibility. The green dots represent the position where p-values are significant at a confidence level α = 0.05.

To assess the putative effect of this mutation on RAD50 function, we performed a protein modeling and simulation analysis. First, we verified the conservation of the residue *NP_005723.2: p.Ala1216* by establishing a multiple sequence alignment using homologous RAD50 proteins of model organisms (Figure 3A). We observed that *Ala1216* is located in a highly conserved segment found in all orthologous sequences belonging to organisms emerging at different evolution periods.

Next, we constructed a homology model of RAD50. The predicted 3D model of hRAD50 consists of the assembly into a homodimer (Figure 3B). Each monomer covers segments 2-221 and 1098-1315 which include a relatively small coiled-coil region and the Walker A and Walker B globular domains. The assembly of the latter contains the Protein-DNA interface and the interaction surface with MRE11. The Stereochemical and the local energy quality were validated for the predicted model in comparison with the template structure (Supplementary Figure 2).

The mutation site is situated in the core of hRAD50 as part of an α helix extended over residues 1203-1221 (Figures 3B,C). Residue A1216 establishes a hydrophobic contact with I1146 situated on another α-helix with a Cα-Cα distance of 5.7 Å. The mutation p.Ala1216Gly is able to preserve this hydrophobic contact. The N-terminal end of the α-helix stabilizes both the Mg^2+^ and the ATPγS particularly, the thiotriphosphate moiety of the substrate. The calculation of ΔΔG_Wt__–__Mut_ predicted a destabilizing effect for all five methods, DynaMut, ENCoM, mCSM, SDM and DUET with values of -0.037, -0.416, -1.4, -2.170, -1.643 kcal.mol^–1^ respectively. Moreover, ENCoM predicted a vibrational entropy energy of 0.52 kcal.mol^–1^.K^–1^ suggesting an increase of the molecular flexibility.

Because a substitution to glycine may affect the flexibility of other regions in the protein, the extent of this mutation was evaluated by simulating hRAD50 using CABS-flex approach (Figure 3D). Our results suggest a significant increase in the RMSF values at a confidence level of 5% (Green dots of Figure 3D) for 71 positions located on both monomers. Of these, residues P165, L166, S1210, I1213 seems to be the most critical as they belong to the helix harboring the mutation (S1210, I1213) or are in the proximity of ATPγS: Mg^2+^ interaction site (P165, L166). Interestingly, the flexibility does also increase for several amino acids that belong to the surface patch involved in the interaction with MRE11 (Seifert et al., 2016). These residues are: F28, L31, V89, N90, G91, K112, T113, L114, E115, G116, I119 of Walker A and D1272, D1295, Q1296, C1297, S1298, K1301, C1302, S1303, V1304 of Walker B.

#### Identification of Novel Candidate Variants

In addition to the 29 genes known to be associated with breast cancer, we investigated all the other variants resulting from WES analysis. In order to identify the relevant variants that could be associated with malignancies, we used several *in silico* prediction tools, genomic databases, biological pathways databases and we performed a literature review. Eleven variants in 11 genes were identified of which 2 candidate variants have been submitted in the ClinVar database. The remaining variants were not reported in the ClinVar database and therefore, their pathogenicity is still not documented. For this, functional and association studies are needed to verify that.

**FIGURE 4:**
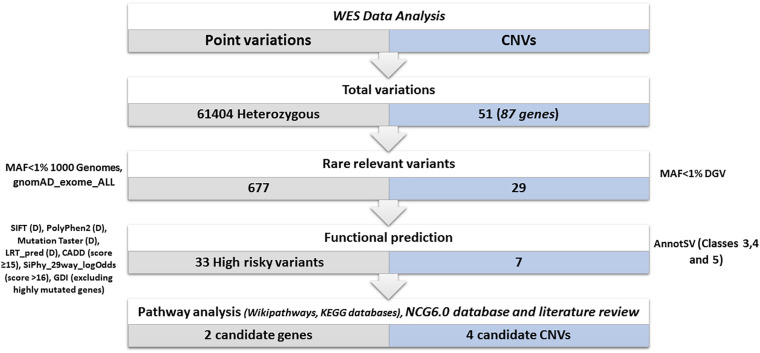
Variants prioritization. Total variations: represents the total number of the identified heterozygous variants and CNVs. Rare relevant variants: we kept (exonic, splicing, non-synonymous, frameshift Indels, stop gain, stop loss) variants. We excluded variants with a frequency > 1% in 1000 genomes and gnomAD_Exome databases and CNVs with a frequency > 1% in DGV. Functional prediction: using several prediction tools: SIFT (Deleterious), PolyPhen2 (Probably damaging), Mutation Taster (disease-causing), LRT_pred (Deleterious), CADD (score ≥ 15), SiPhy_29way_logOdds (score > 16), GDI database (excluding highly mutated genes). Using AnnotSV only CNVs (VUS, likely pathogenic or pathogenic) are kept. Biological pathways investigation and selection of candidate variants and CNVs disrupting cancer genes: Using wikipathways, KEGG databases, network of cancer genes 6.0 database and literature review.

A total of 61,404 heterozygous variants were identified (Figure 4). Among them, 6773 relevant variants (exonic, splicing, non-synonymous, frameshift Indels, stopgain and stoploss) were identified. Variants with MAF > 1% in 1000 Genomes and gnomAD_Exome databases were excluded. Therefore, 677 rare variations were selected for further investigations. In order to select the most relevant SNPs, several *in silico* prediction tools and databases were used as described in the methods section. A list of 33 non-synonymous variants belonging to 33 genes were retained.

These 33 genes were further filtered based on their implication in cancer etiology using Network of Cancer Genes V6.0. The list of genes was compared to those listed in Wikipathways and KEGG datasets as belonging to pathways relevant in cancers. In addition, we performed a literature review to identify genes more likely to be involved in malignancies development.

Based on these analyses, 11 genes were selected, namely *MITF, COL7A1, FOXM1, TSHZ2, AMOTL2, ANKS6, ACACB, LIPE, SNRK, CACNA2D3*, and *SMG1* (Table 2). Among all variants identified on these genes, two variants, namely *ANKS6*_rs199722684 and *MITF*_rs149617956 have been classified in ClinVar as a VUS and a variant with “conflicting interpretations of pathogenicity,” respectively.

**TABLE 2:** Candidate variations identified in whole exome sequenced breast cancer patient.

Chromosome-Position^1^	Gene	Reference sequence	Coding Change	Protein variation	Variant Id	Frequency	Frequency (gnom_AD_exome_ALL)		Prediction tools						Affected pathways	NCG	Literature review
									
					dbSNP	(1000 genomes)		SIFT	Poly phen2	Mutation taster	LRT_pred	CADD_phred	SiPhy_29way_logOdds	ClinVar	Wikipathways/KEGG		
Chr3: 43389157	*SNRK*	NM_001100594	c.1406G > A	p.R469Q	rs3719 00831	−	6.514e-05	D	D	D	D	25.2	17.90	NA	−/−	Cancer gene (Mucosal melanoma)	*SNRK* inhibited colon cancer cell proliferation (Rines et al., 2012)
Chr3: 48602299	*COL7A1*	NM_000094	c.8735G > A	p.C2912Y	−	−	−	D	D	D	D	25.7	18.23	NA	−/Protein digestion and absorption	Cancer gene (Chronic myeloid leukemia)	*COL7A1* is mutated and a candidate tumor suppressor gene in breast cancer. Its hypermethylation in breast cancer is correlated with a poor prognosis (Wood et al., 2007; Chan et al., 2008)
Chr3: 54925426	*CACNA2D3*	NM_018398	c.2195C > T	p.T732M	rs1123 62995	0.003	0.0078	D	D	D	D	34	17.65	NA	MAPK signaling pathway	−	CACNA2D3 was downregulated in esophageal squamous cell carcinoma, nasopharyngeal carcinomas and gliomas (Li et al., 2013; Wong et al., 2013; Jin et al., 2017) and has an antitumor role in endometrial cancer (Kong et al., 2020).
Chr3: 70014091	*MITF*	NM_000248	c.952G > A	p.E318K	rs149 617956	0.0007	0.0014	D	D	D	D	27.9	20.87	Conflicting pathogenicity	RANKL/RANK and Kit receptor signaling pathway/Melanoma, Transcriptional misregulation in cancer, Pathways in cancer	Cancer gene (TNBC)	*MITF* is associated with several cancers. E318K-*MITF* variant is very rare in breast cancer patients (Gromowski et al., 2014)
Chr3: 134090187	*AMOTL2*	NM_001278683	c.263C > T	p.T88M	rs2003 36077	−	0.0001	D	D	D	D	26	17.85	NA	−/Tight junction	−	Amot family members promote the proliferation and invasion of cancer cells, including breast, colon, prostate, cervical, liver, and renal cell cancer (Lv et al., 2017).
Chr9: 101518761	*ANKS6*	NM_173551	c.2267C > T	p.S756L	rs1997 22684	0.0001	0.0005	D	D	D	D	34	16.37	VUs	Ciliary landscape/−	−	SNPs in ANKS6 were nominally significantly associated with breast cancer (Higginbotham et al., 2012)
Chr12: 2968078	*FOXM1*	NM_001243088	c.1973C > T	p.P658L	rs289 19870	0.004	0.0064	D	D	D	D	26.8	16.86	NA	DNA IR-damage and cellular response via ATR, Epithelial to mesenchymal transition in colorectal cancer/Cellular senescence.	Cancer gene (multiple cancers)	Over-expression of FOXM1 is indicative of poor prognosis in breast cancer patients (Bektas et al., 2008)
Chr12: 109625861	*ACACB*	NM_001093	c.2038G > A	p.V680M	rs2015 20813	0.0001	2.438e-05	D	D	D	D	33.0	18.61	NA	AMPK and leptin signaling pathway. Pathways in clear cell renal cell carcinoma VEGFA-VEGFR2 Signaling Pathway/Fatty acid biosynthesis	−	ACACB was downregulated in breast cancer and positively associated with survival time (Bai et al., 2019)
Chr16: 18860605	*SMG1*	NM_015092	c.5557C > G	p.P1853A	rs7720 66609	−	4.875e-05	D	D	D	D	23.2	18.74	NA	−/mRNA surveillance pathway	Cancer gene (multiple cancers)	*SMG1* gene known to be associated with pancreatic cancer risk (Wong et al., 2019)
Chr19: 42914772	*LIPE*	NM_005357	c.1106C > T	p.P369L	rs1385 39064	−	0.0006	D	D	D	D	26.5	16.32	NA	Focal Adhesion-PI3K-Akt-mTOR/AMPK signaling pathway	−	A significant positive correlation with LIPE was observed in BRCA, colorectal and prostate carcinomas) (Nath and Chan, 2016)
Chr20: 51870682	*TSHZ2*	NM_001193421	c.676G > A	p.E226K	rs3755 70590	−	2.031e-05	D	D	D	D	25.5	19.09	NA	−/−	Cancer gene (multiple cancers)	TSHZ2, known to be down-regulated in breast and prostate cancers (Riku et al., 2016)

*LRT_pred_D, Deleterious; Mutation taster_D, Disease causing; NA, Non available; Polyphen2_D, Probably damaging; SIFT_D, Deleterious; VUS, Variant with Uncertain Significance. ^1^Mean chromosome positionis mentioned according to the assembly GRCh37/hg19.*

#### Copy Number Variations Analysis

Copy number variations were also investigated in order to assess their possible contribution to disease susceptibility (Figure 4). Fifty-one CNVs including 30 deletions and 21 duplications were identified with an average size of 24.2 kb (ranging from 64 bp to 123918 bp). Only rare (< 1%) and relevant CNVs (of unknown significance, likely pathogenic or pathogenic and ranked as class 3, class 4, and class 5 respectively based on AnnotSV) were kept (Figure 4).

A total of 7 CNVs affecting 11 genes were identified. Biological pathways analysis has been also performed to select genes linked to breast cancer etiology. In addition, we searched published literature regarding genes affected by CNVs and breast cancer risk (Table 3). Among the 11 genes, 5 had been previously identified as cancer genes or candidate cancer genes namely *TMTC3, BCL3, COL7A1, RNF5*, and *CEP290.*

**TABLE 3:** Candidate rare Copy Number Variations likely associated with cancers.

Chromosome	Start	End	CNV length	CNV type	Genes	Affected Pathways		Network of Cancer genes annotation	DGV Frequency	AnnotSV ranking	Literature review
						Wikipathways	KEGG				
3	48612641	48612972	-331	DEL	*COL7A1*	−	Protein digestion and absorption	Chronic myeloid leukemia	0.00017	4	*COL7A1* gene is mutated and a candidate tumor suppressor gene in breast cancer. Hypermethylation of COL7A1 in breast cancer resulted in loss of ColVII expression in tumors correlating with a poor prognosis (Wood et al., 2007; Chan et al., 2008)
6	32147655	32147903	248	DUP	*RNF5*	−	Protein processing in endoplasmic reticulum	−	−	4	*RNF5* is involved in the control of breast cancer progression and in survival of breast cancer patients (Bromberg et al., 2007). Ubiquitin ligase RNF5 serves an important role in the development of human glioma. RNF5 was significantly associated with diagnosis and prognosis of Hepatocellular carcinoma (Zhu et al., 2016; Gao et al., 2019)
12	88512261	88554006	41745	DUP	*CEP290, TMTC3*	Pathways in clear cell renal cell carcinoma	−	*(TMTC3***)** A candidate cancer gene mutated in Pancreatic cancer	−	4	−
19	45261982	45262872	-890	DEL	*BCL3*	Apoptosis-related network due to altered Notch3 in ovarian cancer	C-type lectin receptor signaling pathway TNF signaling pathway	Hepatobiliary (Cholangiocarcinoma)	−	4	*BCL3* participates in progression of diverse solid tumors (Maldonado and Melendez-Zajgla, 2011)

*AnnotSV ranking: 4 Likely Pathogenic.*

## Discussion

Since their discovery in 1994 and 1995, a wide range of mutational spectrum have been described for *BRCA1* and *BRCA2* genes (Laraqui et al., 2015). Several recurrent mutations have been reported in the Tunisian population. Some of these mutations are specific to Tunisians, others are shared with neighboring populations (Troudi et al., 2008; Mahfoudh et al., 2012; Fourati et al., 2014; Msolly and Kassab, 2015; Riahi et al., 2015). So far, *BRCA1*-c.211dupA mutation has been reported only in Tunisian families with breast cancer (Troudi et al., 2007; Fourati et al., 2014; Riahi et al., 2015). In the present report, 8 carriers of c.211dupA were identified, all originating from the North-East of Tunisia, suggesting that this mutation is specific to this region. This finding is in agreement with the results of Riahi and colleagues where all carriers of the c.211dupA mutation were all originating from the North-Eastern region of Tunisia (Riahi et al., 2015).

In order to unravel the genetic specificities of this mutation and to trace its origin, a haplotype analysis was conducted and results support the founder effect of c.211dupA mutation in the North-East of Tunisia. We have also estimated the *BRCA1-*c.211dupA approximative age. Indeed, it seems that this mutation appeared 130 years ago in Tunisia, during the second half of the 19th century and after the French occupation that took place in 1881. All migratory waves that occurred at the colonial period may be at the origin of new mutations and had therefore a considerable impact on the genetic diversity of the Tunisian population.

The recent origin and the limited geographical distribution of this mutation may also suggest that it could be a *de novo* mutation that occurred in the 19th century in an individual from the North East of Tunisia. Despite the high rates of *de novo* mutation that have been observed in several other genes, only 15 carriers of *de novo BRCA1/2* mutations have been reported to date (Golmard et al., 2016).

Furthermore, investigation of clinicopathological features of c.211dupA carriers showed that the majority exhibited high histological tumor grade and were diagnosed with TNBC. Our findings are in agreement with previous studies reporting that breast carcinomas in *BRCA1* mutation carriers are associated with aggressive tumor characteristics (Armes et al., 1998). Additionally, the TNBC phenotype is the most observed molecular subtype in patients with *BRCA1* mutation and it is well documented that breast cancer patients with *BRCA1* mutation are significantly associated with worse overall survival than patients with a non-mutated *BRCA1* allele (pooled HR = 1.69 (95% CI 1.35 to 2.12, *p* < 0.001; *I*^2^ = 59.1%) (Zhu et al., 2016). Nevertheless, in the present study, median survival of non-metastatic carriers of c.211dupA mutation was 12 years.

We have also investigated the treatment response and the clinical evolution of the disease among carriers. For c.211dupA mutation, two carriers have developed contralateral breast cancer. This observation is similar to those described in the literature where the risk of contralateral recurrence in women with *BRCA* mutation is approximately 40% at 10 years (Metcalfe et al., 2004). Besides, two other carriers have developed ovarian cancer 15 and 13 years after their breast cancer. This observation is also in accordance with what has been reported in the literature on the cumulative risk of developing ovarian carcinoma after breast cancer in *BRCA1* mutation carriers that was estimated to almost 12.7% at 10 years (*p* = 0.03) (Metcalfe et al., 2005). It is noteworthy that personal and/or familial ovarian cancer history was present in a high proportion of families (3/5) carrying the c.211dupA mutation.

Based on the risk of contralateral recurrence and the risk of developing ovarian cancer several years after breast cancer, risk-reducing mastectomy (Hartmann et al., 1999) and salpingo-oophorectomy (Rebbeck et al., 2002) are recommended for *BRCA1*-c.211dupA carriers. Moreover, affected cases carrying this mutation could also benefit from personalized therapeutic options. Indeed, the identification of c.211dupA mutation in a metastatic breast cancer patient helped to reorient her therapeutic decision by using carboplatins, platinum-based agents, that are recommended for the treatment of metastatic *BRCA*-mutated breast cancer with an overall response rate of 80%, including 45% with complete response (Byrski et al., 2012; Turner and Tutt, 2012; Coates et al., 2015).

Therefore, the severe phenotype associated with this mutation (young age of onset, contralateral recurrence, aggressive disease form and ovarian cancer presentation), could be managed if the disease is diagnosed at an early age and if adequate therapeutic and preventive measures are taken.

In the current study, beyond the recurrent founder c.211dupA mutation, a novel private *BRCA1* mutation, c.2418dupA, was identified in a breast cancer patient from the North-East of Tunisia, using targeted gene sequencing. This mutation has never been reported in previous published studies neither in Tunisia nor in other populations. Furthermore, it is not described in the gnomAD database. However, it is listed and classified in ClinVar as pathogenic. This frameshift mutation is located in *BRCA1* exon 11 that binds important homologous recombination proteins including RAD50 and RAD51. Recent studies showed that *BRCA1*-exon 11 constitutes an Ovarian Cancer Clustering Region (OCCR) since mutations located in this region are more associated with ovarian cancer risk than breast cancer (Orr and Savage, 2015; Rebbeck et al., 2015).

Using whole exome sequencing, we also identified a rare exonic VUS on *RAD50* (c.3647C > G, rs1314725075, MAF = 4.061 × 10^–06^) in a breast cancer patient originating also from the North Eastern Tunisian region. Accumulating evidence indicates that *RAD50* is a breast cancer susceptibility gene associated with genomic instability (Heikkinen et al., 2003, 2006; Hsu et al., 2007; Damiola et al., 2014; Kleibl and Kristensen, 2016; Kim et al., 2017; Bian et al., 2019). Indeed, RAD50 is part of the MRE11/RAD50/NBN (MRN) complex that plays a key role in detecting DNA double-strand breaks, recruiting and activating Ataxia-Telangiectasia Mutated protein (ATM) and in processing the DNA repair pathway (Bian et al., 2019; Situ et al., 2019). Mutations in one or more genes forming this complex may lead to hypersensitivity to genotoxic agents and predisposition to malignancy (Bian et al., 2019).

*In silico* analysis at the sequence and structure levels of hRAD50 protein suggest a deleterious impact of the substitution p.Ala1216Gly. The fact that the amino acid Ala is highly conserved in different distantly related orthologous sequences indicates a high positive selective pressure at that position suggesting, therefore, its functional importance for hRAD50. In addition, A1216 is located in the protein core as part of an α-helix segment that stabilizes the binding sites of ATP. Alanine residue, in particular, is the most stabilizing amino acid for helical secondary structures (Serrano et al., 1992; Lopez-Llano et al., 2006). A mutation to glycine, however, would be highly destabilizing for the entire α-helix and consequently for the ATP binding site. Non-capping α-helical Glycine may introduce a significant conformational degree of freedom due to its simple side chain and therefore introduce more flexibility to its local environment. Indeed, Alanine to Glycine mutations were found to have a significant increase in the stabilizing energy of an α helix of 0.4-2 kcal mol^–1^ (Serrano et al., 1992) and in the increase in its local flexibility. Interestingly, the simulation revealed a putative outcome on the interaction of hRAD50 with MRE11 since several residues of the protein-protein interface showed an increase in the local flexibility that need to be investigated further.

Moreover, RAD50 is involved in DNA double-stranded break (DSB) repair by homologous recombination (HR). Recent evidence showed that ovarian cancer patients with a homologous recombination deficiency phenotype (HRD) caused by germline and/or somatic mutations on HR genes exhibit specific clinical behaviors, and improved responses to treatments, such as platinum-based chemotherapy and poly (ADP-ribose) polymerase (PARP) inhibitors (Mateo et al., 2019). However, based on the latest guidelines released by the American Society of Clinical Oncology (ASCO), clinical decisions for ovarian cancer patients should not be based on VUSs identified on HRD genes (Konstantinopoulos et al., 2020).

Therefore, re-evaluating the clinical relevance of VUS identified on HR genes such as the *RAD50* variant characterized in this current study (c.3647C > G) is of keen interest to improve the clinical interpretation of this category of variants and their potential use in precision oncology practice. Indeed, Bope et al., investigated the *in silico* mutation prediction of variants in African genomes and propose recommendations for re-evaluating the pathogenicity of actionable variants used in research and clinical practice (Bope et al., 2019). In addition, Dorschener et al., analyzed actionable pathogenic variants in 500 European and 500 African descent using exome data (Dorschner et al., 2013). The results showed major disparities in Africans with an estimated frequency of around 3.4% for those with European origin and 1.2% for those with African descent. Manrai et al. (2016) reported a study on patients that were misdiagnosed with hypertrophic cardiomyopathy due to lack of access to non-European data. Indeed, patients from African or unspecified ancestry received positive reports with variants misclassified as pathogenic. Authors concluded that the inclusion of even a small number of African-Americans in control cohorts probably would have prevented this misclassification and misdiagnosis. Therefore, based on this evidence and on the results of our current study, we propose to update the classification of the *RAD50* c.3647C > G mutation and maybe reclassifying it as “likely pathogenic variant.”

For this same patient, a missense variant associated with PARP inhibitor response, rs28908468, has been identified on *RAD51B*, which is a paralog of *RAD51* that has an important role in DNA damage response. Indeed, it was demonstrated that RAD51 is a functional biomarker that enables the identification of PARP inhibitors-sensitive breast cancer cases (Castroviejo-Bermejo et al., 2018; Cruz et al., 2018). The *RAD51B* variant was clearly classified by an expert panel in ClinVar as a variant that affects only the drug response. However, for the *RAD50* variant, evidence of the pathogenicity was conflicting. Indeed, expert panel evaluation according to the American College of Medical Genetics and Genomics (ACMG) Standards and Guidelines for the interpretation of sequence variants, showed that this variant is of Uncertain Significance (VUS). Thus, we have made an extensive *in silico* analysis that suggested the need to revise its annotation status.

After investigating known breast cancer genes, we extended our analysis to other genes not yet reported as associated with breast carcinoma. Eleven candidate genes were selected based on several filters, namely: *MITF, COL7A1, FOXM1, TSHZ2, AMOTL2, ANKS6, ACACB, LIPE, SNRK, CACNA2D3*, and *SMG1.* Only two variants identified on these genes were listed in ClinVar including one VUS found on *ANKS6* gene (rs199722684) and a variant of “conflicting interpretations of pathogenicity” identified on *MITF* gene (rs149617956).

Mutations in *ANKS6* are known to cause a nephronophthisis-like phenotype (Taskiran et al., 2014). Based on Cancer Genetic Markers of Susceptibility breast cancer study (CGEMS), 3 SNPs located between *ANKS6* and *GALNT12* were identified as significantly associated with breast cancer (Higginbotham et al., 2012).

*MITF* is associated with development, differentiation, survival and cell cycle regulation. Recent studies showed that the same mutation (rs149617956, E318K) identified on *MITF*, has been reported as associated with a high risk of melanoma, renal cell carcinoma and pancreatic cancer (Gromowski et al., 2014; Sturm et al., 2014). The potential association between E318K and the risk of developing other malignancies such as breast carcinoma has been evaluated by Gromowski et al. Authors concluded that E318K is very rare in breast cancer patients (Gromowski et al., 2014).

Here we also identified 4 candidate CNVs disrupting 5 genes associated with several carcinomas and involved in interesting biological pathways namely *BCL3, COL7A1, RNF5, CEP290* and *TMTC3.* The screening of these CNVs could be considered particularly in breast cancer patients’ non-carriers of pathogenic point mutations.

## Conclusion

The identification of founder and novel private mutations on *BRCA1/2* as well as functional variants on genes involved in the homologous recombination pathway has significant impact on genetic screening and clinical management of breast and ovarian cancer patients. Our findings also highlight the importance of the use of NGS technologies in detecting novel mutations involved in breast cancer susceptibility in under-investigated populations. *In silico* analyses that were performed in this study on *RAD50*-c.3647C > G variant classified as VUS in ClinVar suggests a re-evaluation of the current classifications of several variants in public databases. Such a reconsideration would improve criteria of pathogenicity assessment by studying African populations, in particular, North Africans that are still poorly investigated.

## Data Availability Statement

The datasets for this article are not publicly available due to concerns regarding participant/patient anonymity. Requests to access the datasets should be directed to the corresponding author.

## Ethics Statement

The study was conducted according to the declaration of Helsinki and with the approval of the biomedical ethics committee of Institut Pasteur de Tunis (2017/16/E/Hôpital A-M). Written informed consent was obtained from the participants in this study for the publication of any potentially identifiable images or data included in this article.

## Author Contributions

NaM did the experiments, analysis, and data interpretation, and drafted the manuscript. YH and MB did analysis and data interpretation, and reviewed the manuscript. MBR contributed in data analysis. SBN, HE, NeM, SL, JA, OJ, HanB, RM, AH, and KR contributed to clinical investigation of patients. HO performed the *in silico* analyses and reviewed the manuscript. YH and SA contributed to study concept and design. YH, HamB, SB, and SA critically revised the manuscript. YH and SA supervised the study. All authors read and approved the final manuscript.

## Conflict of Interest

The authors declare that the research was conducted in the absence of any commercial or financial relationships that could be construed as a potential conflict of interest.

## Footnotes

1 www.ins.nat.tn

## Supplementary Material

Supplementary Figure 1 Diagram of the Short Tandem Repeats used for the haplotype analysis. This map is based on the information provided by UniSTS, UCSC. The location and order of the loci used in the present study, as well as their physical and genetic positions are shown. These data are extracted from the Marshfield genetic map (Marshfield Center for Medical Genetics).

Supplementary Figure 2 Assessment of the stereochemical **(A)** and local **(B)** qualities of hRAD50 homology model in comparison with the template structure by establishing the Ramachandran Plot and the Verif3D profile respectively.

## Abbreviations

ACMG: American College of Medical Genetics and Genomics
ATM: ataxia-telangiectasia mutated protein
CNVs: copy number variations
DNA: deoxyribonucleic acid
DSB: double-stranded break
HER2: human epidermal growth factor receptor 2
HR: homologous recombination
HRD: homologous recombination deficiency phenotype
IDC: intraductal carcinoma
InDels: insertions and deletions
INS: Institute of Statistics
KEGG: kyoto encyclopedia of genes and genomes
MAF: minor allele frequency
MRN: MRE11/RAD50/NBN
NCG: network of cancer genes
NGS: next generation sequencing
OCCR: ovarian cancer clustering region
PARP: poly (ADP-ribose) polymerase
PCR: polymerase chain reaction
PDB: protein data bank
RMSF: root mean square fluctuation
SBR: Scarff-Bloom and Richardson
SNP: single nucleotide polymorphism
SV: structural variations
TGS: target gene sequencing
TNBC: triple negative breast cancer
VarAFT: variant annotation and filtering tool
VUS: variant of unknown significance
WES: whole exome sequencing.
