# Supplementary figures and images for: Identification of Novel BRCA1 and RAD50 Mutations Associated With Breast Cancer Predisposition in Tunisian Patients
Source: Front Genet. 2020 Nov 6;11:552971. doi: 10.3389/fgene.2020.552971 (PMC7677579; doi:10.3389/fgene.2020.552971)

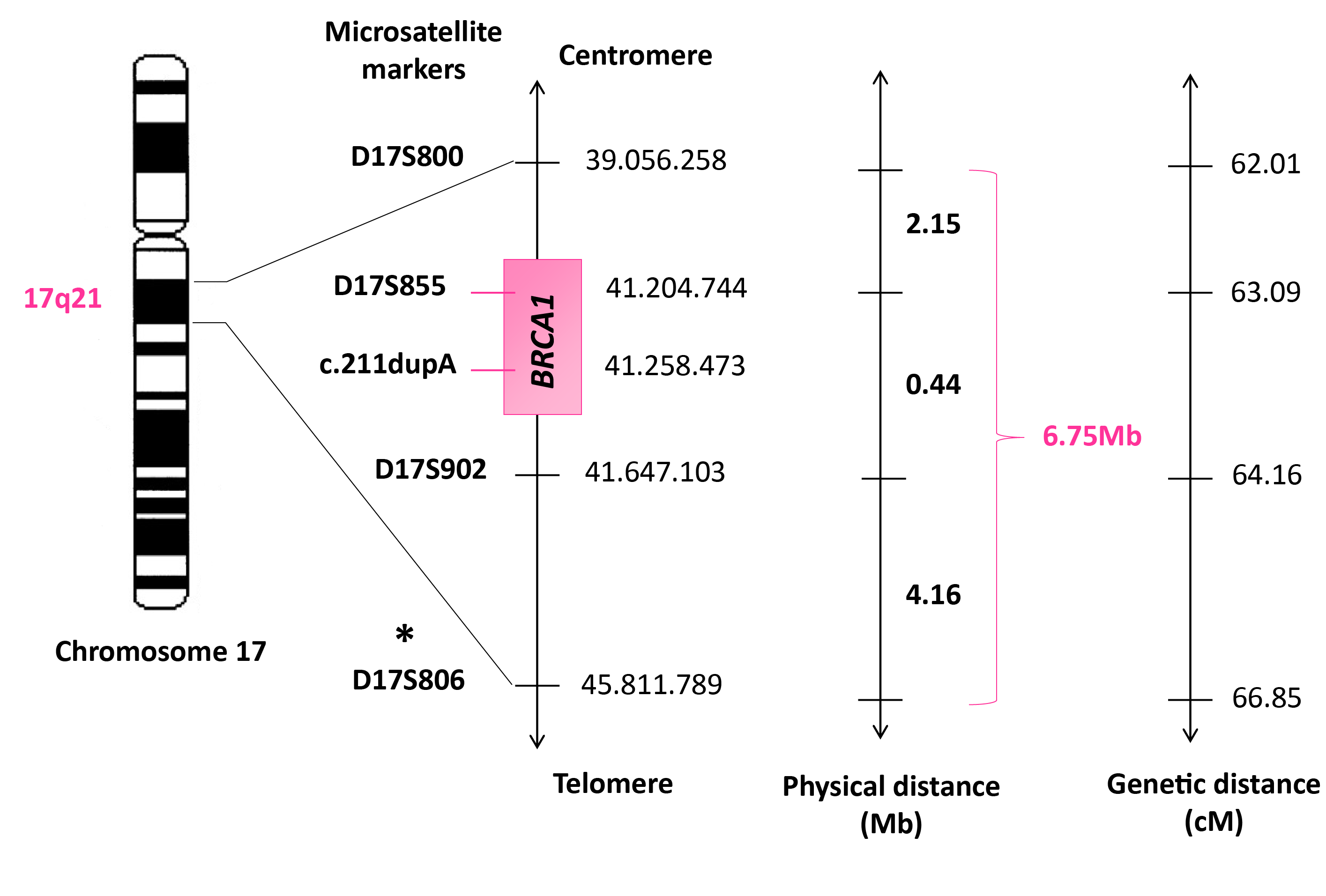

Supplement: Supplementary Figure 1 — Diagram of the Short Tandem Repeats used for the haplotype analysis. This map is based on the information provided by UniSTS, UCSC. The location and order of the loci used in the present study, as well as their physical and genetic positions are shown. These data are extracted from the Marshfield genetic map (Marshfield Center for Medical Genetics). [file Image_1.TIF]

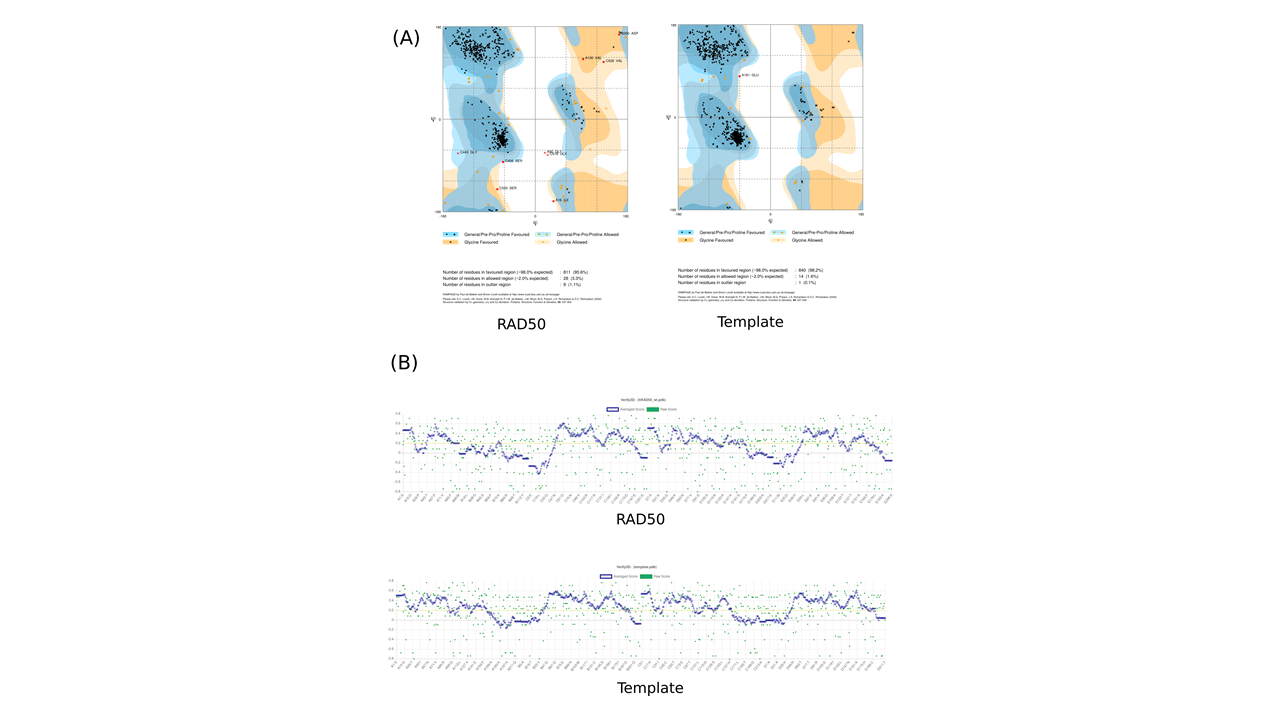

Supplement: Supplementary Figure 2 — Assessment of the stereochemical (A) and local (B) qualities of hRAD50 homology model in comparison with the template structure by establishing the Ramachandran Plot and the Verif3D profile respectively. [file Image_2.TIF]
