# Supplementary material for: Identification of Novel BRCA1 and RAD50 Mutations Associated With Breast Cancer Predisposition in Tunisian Patients
Source: Front Genet. 2020 Nov 6;11:552971. doi: 10.3389/fgene.2020.552971 (PMC7677579; doi:10.3389/fgene.2020.552971)
Supplement: Supplementary file 3 [file Table_1.DOCX]

**Supplementary Table S1. Coverage statistics of WES data**

| Coverage Data | BC-TN-F0019 |
| --- | --- |
| Total reads | 49 111 123 |
| % Reads mapped to human genome | 99.64 |
| Total coverage | 2 718 453 424 |
| Mean read depth of target regions (X) | 52.74 |
| % Coverage of target regions (more than 10X) | 91.3 |
| % Coverage of target regions (more than 20X) | 82.7 |
| % Coverage by genes | |
| *High penetrance genes* | |
| *BRCA1* | 99.8 |
| *BRCA2* | 99.44 |
| *CDH1* | 96.19 |
| *PTEN* | 98.43 |
| *STK11* | 91.78 |
| *TP53* | 98.35 |
| *Moderate penetrance genes* | |
| *ATM* | 95.53 |
| *CHEK2* | 82.06 |
| *BRIP1* | 95.52 |
| *PALB2* | 100 |
| *Other susceptibility genes* | |
| *BARD1* | 100 |
| *BLM* | 100 |
| *FAM175A* | 97.48 |
| *FANCC* | 97.7 |
| *FANCM* | 92.84 |
| *MAPKAP1* | 98.69 |
| *MLH1* | 97.93 |
| *MRE11A* | 83.97 |
| *MSH2* | 96.17 |
| *NBN* | 94.66 |
| *NF1* | 88.35 |
| *PMS2* | 78.87 |
| *RAD50* | 90.35 |
| *RAD51B* | 90.6 |
| *RAD51C* | 96.91 |
| *RAD51D* | 94.46 |
| *RECQL* | 99.38 |
| *RINT1* | 95.12 |
| *XRCC2* | 96.44 |
