# Supplementary material for: Identification of Novel BRCA1 and RAD50 Mutations Associated With Breast Cancer Predisposition in Tunisian Patients
Source: Front Genet. 2020 Nov 6;11:552971. doi: 10.3389/fgene.2020.552971 (PMC7677579; doi:10.3389/fgene.2020.552971)
Supplement: Supplementary file 4 [file Table_2.DOCX]

**Supplementary Table S2. Exonic-splicing variants on known hereditary breast and ovarian cancer genes**

| Genes | Chromosome | Position | Variant ID | Sequence variation | Frequency  (1000Genomes) | Frequency (gnom_AD_Exome_ALL) | | Localization | | ClinVar |
| --- | --- | --- | --- | --- | --- | --- | --- | --- | --- | --- |
| *ATM* | No detected variants | | | | | | | | | |
| *BARD1* |  | | | | | | | | | |
|  | Chr2 | 215645545 | rs2070096 | c.1053G>C | 0.190 | 0.2061 | Exonic | | Benign | |
| *BLM* |  | | | | | | | | | |
|  | Chr15 | 91337479 | rs2227933 | c.3102G>A | 0.151 | 0.1561 | Exonic | | Benign | |
|  |  | 91346923 | rs2227934 | c.3531C>A | 0.133 | 0.1514 | Exonic | | Benign | |
|  |  | 91354505 | rs1063147 | c.3945C>T | 0.132 | 0.1515 | Exonic | | Benign | |
| *BRCA1* |  | | | | | | | | | |
|  | Chr17 | 41245471 | rs4986850 | c.2077G>A | 0.0335 | 0.0587 | Exonic | | Benign | |
|  |  | 41245466 | rs1799949 | c.2082C>T | 0.336 | 0.3533 | Exonic | | Benign | |
|  |  | 41245237 | rs16940 | c.2311T>C | 0.335 | 0.3482 | Exonic | | Benign | |
|  |  | 41244936 | rs799917 | c.2612C>T | 0.543 | 0.4032 | Exonic | | Benign | |
|  |  | 41244435 | rs16941 | c.3113A>G | 0.335 | 0.3490 | Exonic | | Benign | |
|  |  | 41244000 | rs16942 | c.3548A>G | 0.352 | 0.3534 | Exonic | | Benign | |
|  |  | 41234470 | rs1060915 | c.4308T>C | 0.336 | 0.3493 | Exonic | | Benign | |
|  |  | 41223094 | rs1799966 | c.4837A>G | 0.355 | 0.3546 | Exonic | | Benign | |
| *BRCA2* |  | | | | | | | | | |
|  | Chr13 | 32912299 | rs543304 | c.3807T>C | 0.168 | 0.1747 | Exonic | | Benign | |
| *BRIP1* |  | | | | | | | | | |
|  | Chr17 | 59760996 | rs4986763 | c.3411T>C | 0.620 | 0.5973 | Exonic | | Benign | |
|  |  | 59763347 | rs4986764 | c.2755T>C | 0.627 | 0.5998 | Exonic | | Benign | |
|  |  | 59763465 | rs4986765 | c.2637A>G | 0.815 | 0.7159 | Exonic | | Benign | |
| *CDH1* |  | | | | | | | | | |
|  | Chr16 | 68857441 | rs1801552 | c.2076T>C | 0.718 | 0.6500 | Exonic | | Benign | |
| *CHEK2* | No detected variants | | | | | | | | | |
| *FAM175A* | No detected variants | | | | | | | | | |
| *FANCC* | No detected variants | | | | | | | | | |
| *FANCM* |  | | | | | | | | | |
|  | Chr14 | 45605463 | rs61746895 | c.229A>G | 0.016 | 0.0114 | Exonic | | Benign | |
|  |  | 45606287 | rs10138997 | c.524C>T | 0.205 | 0.1011 | Exonic | | Benign | |
|  |  | 45644589 | rs1367580 | c.2554G>T | 0.232 | 0.1490 | Exonic | | Benign | |
|  |  | 45669205 | rs8018014 | c.6063T>C | 0.028 | 0.0138 | Exonic | | Benign | |
| *MAPKAP1* | No detected variants | | | | | | | | | |
| *MLH1* | No detected variants | | | | | | | | | |
| *MRE11A* | No detected variants | | | | | | | | | |
| *MSH2* | No detected variants | | | | | | | | | |
| *NBN* |  | | | | | | | | | |
|  | Chr8 | 90958422 | rs1061302 | c.1770A>G | 0.352 | 0.3470 | Exonic | | Benign | |
|  |  | 90967711 | rs709816 | c.951T>C | 0.608 | 0.4640 | Exonic | | Benign | |
|  |  | 90990479 | rs1805794 | c.307G>C | 0.357 | 0.3472 | Exonic | | Benign | |
|  |  | 90965828 | rs3026268 | c.1243A>G | 0.008 | 0.0017 | Exonic | | Benign | |
| *NF1* | No detected variants | | | | | | | | | |
| *PALB2* | No detected variants | | | | | | | | | |
| *PMS2* |  | | | | | | | | | |
|  | Chr7 | 6026988 | rs1805321 | c.1408C>T | 0.358 | 0.3879 | Exonic | | Benign | |
|  |  | 6036980 | rs1805319 | c.780C>G | 0.831 | 0.8015 | Exonic | | Benign | |
|  |  | 6037058 | rs60794673 | c.706-4T>- | 0.453 | 0.4310 | Splicing | | Benign | |
|  |  | 6013049 | rs1802683 | c.2570G>C | **-** | 0.2856 | Exonic | | Benign | |
| *PTEN* | No detected variants | | | | | | | | | |
| *RAD50* |  | | | | | | | | | |
|  | **Chr5** | **131976392** | **rs1314725075** | **c.3647C>G** | **-** | **4.061e-06** | **Exonic** | | **VUS** | |
| *RAD51B* |  | | | | | | | | | |
|  | **Chr14** | **69061259** | **rs28908468** | **c.1094C>G** | **0.019** | **0.0295** | **Exonic** | | **Drug Response** | |
| *RAD51C* | No detected variants | | | | | | | | | |
| *RAD51D* |  | | | | | | | | | |
|  | Chr17 | 33433487 | rs4796033 | c.554G>A | 0.095 | 0.1566 | Exonic | | Benign | |
| *RECQL* | No detected variants | | | | | | | | | |
| *RINT1* | No detected variants | | | | | | | | | |
| *STK11* | No detected variants | | | | | | | | | |
| *TP53* | No detected variants | | | | | | | | | |
| *XRCC2* | No detected variants | | | | | | | | | |
